# Safety and efficacy analysis of neoadjuvant pertuzumab, trastuzumab and standard chemotherapy for HER2–positive early breast cancer: real–world data from NeoPowER study

**DOI:** 10.1186/s12885-024-12506-0

**Published:** 2024-06-15

**Authors:** Fabio Canino, Monica Barbolini, Ugo De Giorgi, Tommaso Fontana, Valeria Gaspari, Caterina Gianni, Lorenzo Gianni, Antonio Maestri, Santino Minichillo, Luca Moscetti, Antonella Mura, Stefania Vittoria Luisa Nicoletti, Claudia Omarini, Rachele Pagani, Samanta Sarti, Angela Toss, Claudio Zamagni, Riccardo Cuoghi Costantini, Federica Caggia, Giuseppina Antonelli, Federica Baglio, Lorenzo Belluzzi, Giulio Martinelli, Salvatore Natalizio, Ornella Ponzoni, Massimo Dominici, Federico Piacentini

**Affiliations:** 1 Division of Medical Oncology, Department of Medical and Surgical Sciences for Children and Adults, University Hospital of Modena, Largo del Pozzo 71, Modena, 41124 Italy; 2 Division of Medical Oncology, Department of Oncology and Hematology, University Hospital of Modena, Modena, Italy; 3 Department of Medical Oncology, IRCCS Istituto Romagnolo per lo Studio dei Tumori (IRST) “Dino Amadori”, Meldola, Italy; 4 Istituto di Ricovero e Cura a Carattere Scientifico (IRCCS) Azienda Ospedaliero-Universitaria di Bologna, Bologna, Italy; 5 Department of Medical Oncology, Infermi Hospital, AUSL della Romagna, Rimini, Italy; 6 Department of Medical Oncology, AUSL di Bologna, Bologna, Italy; 7 Unit of Clinical Statistics, University Hospital of Modena, Modena, Italy

**Keywords:** Neoadjuvant treatment, HER2+, Early breast cancer, Pertuzumab, HER2 dual blockade, Real world data

## Abstract

**Background:**

The addition of pertuzumab (P) to trastuzumab (H) and standard chemotherapy (CT) as neoadjuvant treatment (NaT) for patients with HER2 + breast cancer (BC), has shown to increase the pathological complete response (pCR) rate, without main safety concerns. The aim of NeoPowER trial is to evaluate safety and efficacy of *P* + H + CT in a real–world population.

**Methods:**

We retrospectively reviewed the medical records of stage II–III, HER2 + BC patients treated with NaT: who received *P* + H + CT (neopower group) in 5 Emilia Romagna institutions were compared with an historical group who received H + CT (control group). The primary endpoint was the safety, secondary endpoints were pCR rate, DRFS and OS and their correlation to NaT and other potential variables.

**Results:**

260 patients were included, 48% received *P* + H + CT, of whom 44% was given anthraciclynes as part of CT, compared to 83% in the control group. The toxicity profile was similar, excluding diarrhea more frequent in the neopower group (20% vs. 9%). Three patients experienced significant reductions in left ventricular ejection fraction (LVEF), all receiving anthracyclines. The pCR rate was 46% (*P* + H + CT) and 40% (H + CT) (*p* = 0.39). The addition of P had statistically correlation with pCR only in the patients receiving anthra-free regimens (OR = 3.05,*p* = 0.047). Preoperative use of anthracyclines (OR = 1.81,*p* = 0.03) and duration of NaT (OR = 1.18,*p* = 0.02) were statistically related to pCR. 12/21 distant-relapse events and 14/17 deaths occurred in the control group. Patients who achieve pCR had a significant increase in DRFS (HR = 0.23,*p* = 0.009).

**Conclusions:**

Adding neoadjuvant P to H and CT is safe. With the exception of diarrhea, rate of adverse events of grade > 2 did not differ between the two groups. P did not increase the cardiotoxicity when added to H + CT, nevertheless in our population all cardiac events occurred in patients who received anthracycline-containing regimens. Not statistically significant, higher pCR rate is achievable in patients receiving neoadjuvant *P* + H + CT. The study did not show a statistically significant correlation between the addition of P and long-term outcomes.

**Supplementary Information:**

The online version contains supplementary material available at 10.1186/s12885-024-12506-0.

## Background

Breast cancer is the most frequently diagnosed malignancy and the leading cause of cancer death in women in Italy, with about 55,000 new diagnoses and 12,500 deaths annually [1].

About 15–20% of invasive breast cancers overexpress human epidermal growth factor 2 (HER2). HER2 positive (HER2+) breast cancer is independently associated with high grade, aggressive phenotype, and poorer prognosis, compared to HER2 negative (HER2−) counterpart [2].

The development of anti-HER2 agents has resulted in a deep improvement in the outcome of patients with this type of disease. In particular, the addition of the monoclonal antibody trastuzumab (H) to standard neoadjuvant chemotherapy (CT) regimens increased pathological complete response (pCR) rates, reducing the risk of relapse [3].

pCR is defined as the absence of residual invasive disease in the breast and axillary lymph nodes (excluding carcinoma in situ) after preoperative treatment. pCR has long been used as a surrogate for long-term efficacy outcomes in neoadjuvant studies. In a pooled analysis, Cortazar et al. [4] demonstrated that patients who obtained pCR after preoperatory treatment for breast cancer, have an improvement in event-free survival (EFS) and overall survival (OS) compared to those who obtained a no-pCR. This correlation is strongest for the most aggressive breast cancer phenotypes, triple negative and HER2+.

Subsequent studies have shown that adding pertuzumab (P) to H and neoadjuvant CT further increased pCR rates. The greater effectiveness of the dual HER2 blockade is due to the synergistic action of the two monoclonal antibodies, which bind different epitopes of the HER2 receptor: H inhibits ligand-independent signaling and induces antibody dependent cellular cytotoxicity - ADCC; on the other hand, P inhibits ligand-dependent heterodimerization with other members of the HER family. The final effect is a more powerful inhibition of the proliferation of cancer cells and an increase in apoptosis [5].

Neosphere [6, 7], TRYPHAENA [8, 9] and Berenice [10] trials evaluated the efficacy and safety of adding P to H and neoadjuvant chemotherapy, showing higher rates of pCR that correlated with improved long-term outcomes (progression free survival – PFS, disease free survival – DFS), without worsening treatment tolerability and potential cardiotoxicity.

In particular, the results of NeoSphere and TRYPHAENA guaranteed the accelerated approval for the use of P in the neoadjuvant setting by the FDA and EMA in 2013. Nevertheless, the drug is refundable by the Italian healthcare system in this setting only from November 2023, so to prescribe it was necessary to require nominal use for each patient.

The aim of the NeoPower study was to collect and analyze the data of patients with HER2 + early breast cancer (eBC) treated in the neoadjuvant setting with P, H and chemotherapy in different cancer centers in Emilia Romagna, in order to evaluate the tolerability and efficacy of the treatment in real life.

## Patients and methods

### Study design and participants

NeoPowER was an observational, retrospective, multicenter study that involved patients treated at the following cancer centers in Emilia Romagna: AOU Policlinico di Modena; AUSL Bologna, Ospedale Bellaria; IRCCS Istituto Romagnolo per lo Studio dei Tumori (IRST) Dino Amadori di Meldola; AUSL della Romagna, Ospedale Infermi di Rimini; AOU Bologna, IRCCS Policlinico Sant’Orsola-Malpighi.

The study included: patients aged 18 years or older and baseline Eastern Cooperative Oncology Group (ECOG) performance status of 0 or 1; with operable (T2-3, N0-1, M0), locally advanced or inflammatory (T2 3, N2-3, M0 or T4a-d, any N, M0) breast cancer; HER2 overexpression confirmed by immunohistochemistry (IHC) 3 + or 2 + and amplified in situ hybridization (ISH), as per local laboratory assessment; who received at least one and no more than eight course of NaT with anti HER2 agents as clinical practice (patients enrolled in any clinical trials were excluded), followed by adequate surgical treatment on T and N.

Main exclusion criteria were: metastatic disease (stage IV) at diagnosis; HER2 negative breast cancer (HER2 score 0, 1 + or 2 + and ISH negative); neoadjuvant treatment other than that considered in this study; failure to perform surgery after neoadjuvant treatment due to patient refusal, evidence of metastatic disease or other reasons.

Patients who received pertuzumab, trastuzumab and chemotherapy formed the *P* + H + CT (or Neopower) group, while those treated at Modena cancer centre with trastuzumab and chemotherapy constituted the H + CT (or control) group.

The primary endpoint was the safety of neoadjuvant treatment. The main adverse events were graded according to the National Cancer Institute Common Terminology Criteria for Adverse Events (CTCAE) version 5.0.

Secondary endpoints were: pCR rate (pCR defined as absence of residual invasive neoplastic cells at microscopic examination of the breast and axillary lymph nodes after surgery. The presence of isolated tumour cells – ITCs, was not considered pCR); distant relapse free survival – DRFS (the time from the first date of no disease [i.e date of surgery] to the first documentation of distant relapsed disease / last follow-up); overall survival – OS (the time from the date of diagnosis to death / last follow-up).

### Data collection and procedures

Clinicopathological data were acquired from electronic medical records of each centres and included: patient demographics; tumor size “T” (determined preferably with magnetic resonance imaging - RMI, alternatively with ultrasound and/or mammography), nodal status “N”, stage (according to TNM classification, 8th edition), grade, biological characteristics including hormone receptor expression (estrogen and progesterone receptors – ER/PgR – positivity was defined as ≥ 1% cells staining by IHC), Ki67 and HER2score before and after neoadjuvant treatment and surgery; type of chemotherapy used, duration and main adverse events of neoadjuvant treatment; type of surgery and adjuvant treatments performed according to clinical practice (anti HER2 agents, chemotherapy, endocrine therapy, radiotherapy).

Pertuzumab was administred at loading dose of 840 mg, followed by 420 mg every 21 days; trastuzumab loading dose was 8 mg/kg, followed by 6 mg/kg every 21 days (or 4 mg/kg loading dose followed by 2 mg/kg weekly). The choice of the taxane-based (docetaxel or paclitaxel) chemotherapy regimen was at the physician’s discretion. Changes in dose, schedule and drugs for toxicities were carried out according to standard guidelines.

As per clinical practice, all patients underwent echocardiogram at the beginning, before anthacycline therapy and at the end of neoadjuvant treatment. The values of left ventricular ejection fraction (LVEF) by the echocardiograms at each timepoint were registered to analyze cardiac safety. In patients without specific symptoms, we considered a decrease in LVEF of 10–15% from baseline and < 50% or ≥ 16% from baseline (regardless of the value achieved) to be significant.

This observational research was reported according to STROBE guidelines (https://www.strobe-statement.org/), while the checklists were reported as supplemental materials.

### Ethical committee

This study was performed in line with the principles of the Declaration of Helsinki. Approval was granted by the Ethics Committee of the Area Vasta Emilia Nord (approval date 05/03/2019, approval code 1133/2018). All individual participants included in the study accepted and signed the informed consent form for the treatment and publication of their anonymized clinical data. Data were analysed in aggregate and anonymous form.

### Statistical analysis

Continuous variables were reported as median value with interquartile range (IQR) or mean and standard deviation (SD), while categorical variables were reported as absolute and percentage frequencies.

Comparative assessments were performed by applying Pearson’s χ2 test or Fisher exact test for categorical data and Student t test or Wilcoxon-Mann-Whitney test for continuous variables.

Univariable and multivariable logistic regression models were used to assess the impact of study arms and covariates on pCR.

DRFS and OS was calculated using Kaplan-Meier estimators and comparisons between curves were performed with the Mantel-Cox log-rank test.

Cox proportional hazard regression models were used to estimate hazard ratios (HR) and their 95% CIs and *p*-values. Multivariable Cox regression models have also been defined in order to take into account the possible effect of other covariates.

The covariates inclusion in all multivariables regression models was driven by both their clinical relevance and the imbalances emerged from the univariable analysis.

For all analyses, the results were considered statistically significant when associated with a *p*-value below the significance level alpha 0.05.

All analyses were carried out using R statistical software version 4.2.1 (The R Foundation for Statistical Computing, 2022).

## Results

### Patient and treatment characteristics

The study included 260 elegible patients. We retrospectively reviewed the electronic medical records of 126 patients (48%) who received pertuzumab, trastuzumab and chemotherapy (*P* + H + CT or neopower group) in 5 Emilia Romagna oncology centers (Modena, Bologna Bellaria, Bologna S. Orsola, Meldola, Rimini) from May 2016 to October 2022. The data of 134 patients (52%) who received trastuzumab and chemotherapy (H + CT or control group) at Modena Cancer Center between January 2007 and July 2021, were collected as control group.

The characteristics of patients (Table 1) were well balanced. Median age was 52 years in both groups, cN0 at diagnosis in about 36–40% of patients, stage II in 73% and hormone receptors positive (HR+) in 61%. In contrast, 62% of patients in the *P* + H + CT cohort had a Ki67 ≥ 30%, compared to 43% in H + CT group.

**Table 1:** Patients’demographic and pathological data

	*P* + H + CT NEOPOWER Group *n* = 126 (%)		H + CT CONTROL Group *n* = 134 (%)	
**Median age**				
	52	[28–76]	51,5	[28–84]
**Median BMI**				
	25	[17–47]	24,6	[16–42]
**Menopause**				
No	49	(38,9%)	64	(47,8%)
Yes	62	(49,2%)	68	(50,7%)
Unknown	15	(11,9%)	2	(1,5%)
**ECOG PS**				
0	64	(50,8%)	111	(82,8%)
1	2	(1,6%)	23	(17,2%)
Unknown	60	(47,6%)	0	(0,7%)
**Istology**				
Ductal	116	(92,1%)	127	(94,8%)
Lobular	3	(2,4%)	6	(4,5%)
Others	8	(6,3%)	1	(0,7%)
**Clinical lymphnode (cN)**				
0	50	(39,7%)	48	(35,8%)
1–3	76	(60,3%)	86	(64,2%)
**Stage**				
II	92	(73,0%)	99	(73,9%)
III	34	(27,0%)	35	(26,1%)
**Grade**				
2	29	(23,0%)	22	(16,4%)
3	85	(67,5%)	102	(76,1%)
Unknown	12	(9,5%)	10	(7,5%)
**HR status**				
HR negative	49	(38,9%)	51	(38,1%)
HR positive	77	(61,1%)	83	(61,9%)
**Ki67 cutoff**				
< 30	48	(38,1%)	73	(54,5%)
≥ 30	78	(61,9%)	58	(43,3%)
Unknown	0		3	(2,2%)

All the patients received standard taxanes based neoadjuvant CT associated to anti-HER2 agents. CT backbones were as follow: in the *P* + H + CT cohort, 63% of patients received docetaxel (D) and 44% sequential anthracyclines. In the control group, weekly paclitaxel (wPtx) was administered in 93% of cases, anthracycline-containing regimens was given to 83% of patients, and among them, 44% also received 5-fluorouracil (Table 2).

**Table 2:** Neodjuvant treatment data

	*P* + H + CT NEOPOWER Group *n* = 126 (%)		H + CT CONTROL Group *n* = 134 (%)	
**Median NaT Duration (days)**				
	116	[11–226]	153	[14–210]
**Nat Duration Cut off (cycles)**				
≤ 4 cycles	49	(38,9%)	21	(15,7%)
5–8 cycles	77	(61,1%)	113	(84,3%)
**Anthracyclines administration**				
NO	70	(55,6%)	23	(17,2%)
YES	56	(44,4%)	111	(82,8%)
**Type of Taxane**				
Paclitaxel (Ptx)	37	(29,4%)	125	(93,3%)
Docetaxel (D)	79	(62,7%)	5	(3,7%)
Switch (D→Ptx)	9	(7,1%)	4	(3,0%)
None	1^#^	(0,8%)	0	
**N° Taxanes administrations**				
≤ 4 taxanes cycles	103	(81,7%)	132	(98,5%)
5–6 taxanes cycles	23	(18,3%)	2	(1,5%)
**Fluorouracil administration**				
NO	126	(100,0%)	75	(56,0%)
YES	0		59	(44,0%)
**NAT scheme**				
Docetaxel (D)	53	(42,1%)	1	(0,7%)
Paclitaxel (Ptx)	10	(7,9%)	21	(15,8%)
Switch (D→Ptx)	5	(4,0%)	1	(0,7%)
EC – Paclitaxel	20	(15,9%)	15	(11,2%)
Paclitaxel - (F)EC	11	(8,7%)	92	(68,6%)
Docetaxel - (F)EC	25	(19,8%)	4	(3,0%)
Carbo Docetaxel	1	(0,8%)	0	
None	1^1^	(0,8%)	0	

#1 patient in Neopower group did not receive any taxane infusion during NAT

The median time to surgery and the number of mastectomies performed were similar in the two cohorts. Instead, more axillary lymph node dissections – ALND – were performed in the control group, 69% vs. 34%.

With regard to post-operative treatment, 7% (H + CT) vs. 29% (*P* + H + CT) of patients received anthracyclines as adjuvant chemotherapy. In the Neopower group, 12% and 28% of patients received H + P and trastuzumab-emtansine (TDM1) respectively as post-neoadjuvant treatment, while more than 87% recevived H alone in the control group (Table 3).

**Table 3:** pCR rate and post-NaT data (surgical and adjuvant treatments)

	*P* + H + CT NEOPOWER Group *n* = 125* (%)		H + CT CONTROL Group *n* = 134 (%)	
**Time to surgery cut off**				
≤ 28 days	55	(44,0%)	81	(60,4%)
> 28 days	70	(56,0%)	51	(38,1%)
Unknown	0		2	(1,5%)
**Type of surgery (breast)**				
Lumpectomy	68	(54,4%)	67	(50,0%)
Mastectomy	56	(44,8%)	67	(50,0%)
None	3	(2,4%)	0	
**Type of surgery (lymphnodes)**				
SLNB	76	(60,8%)	41	(30,6%)
ALND	43	(34,4%)	93	(69,4%)
SLNB → ALND	6	(4,8%)	3	(2,2%)
None	1	(0,8%)	0	
**pCR**				
NO	68	(54,4%)	80	(59,7%)
YES	57	(45,6%)	54	(40,3%)
**Adjuvant CT**				
NO	88	(70,4%)	125	(93,3%)
YES	36	(28,8%)	9	(6,7%)
Unknown	1	(0,8%)	0	
**Adjuvant HER2 inhibitor**				
H	73	(58,4%)	117	(87,3%)
H + P	15	(12,0%)	0	
TDM1	35	(28,0%)	7	(5,2%)
None	0		10	(7,5%)
Unknown	2	(1,6%)	0	
**Adjuvant OT**				
NO	49	(39,2%)	50	(37,3%)
YES	75	(60,0%)	84	(62,7%)
Unknown	1	(0,8%)	0	
**Adjuvant RT**				
NO	29	(23,2%)	31	(23,1%)
YES	94	(75,2%)	103	(76,9%)
Unknown	2	(1,6%)	0	

* 1 patient in the neopower group was excluded from the PCR and survival analysis for prematurely stopping preoperative treatment due to toxicity

### Safety analysis

252 patients were included in the safety analysis, 123 (49%) received P. There were 729 treatment-related adverse events (AEs) of any grade (G), 328 and 401 in neopower and control respectively. Overall, 88% AEs were G1-2 in both groups.

**Fig. 1:**
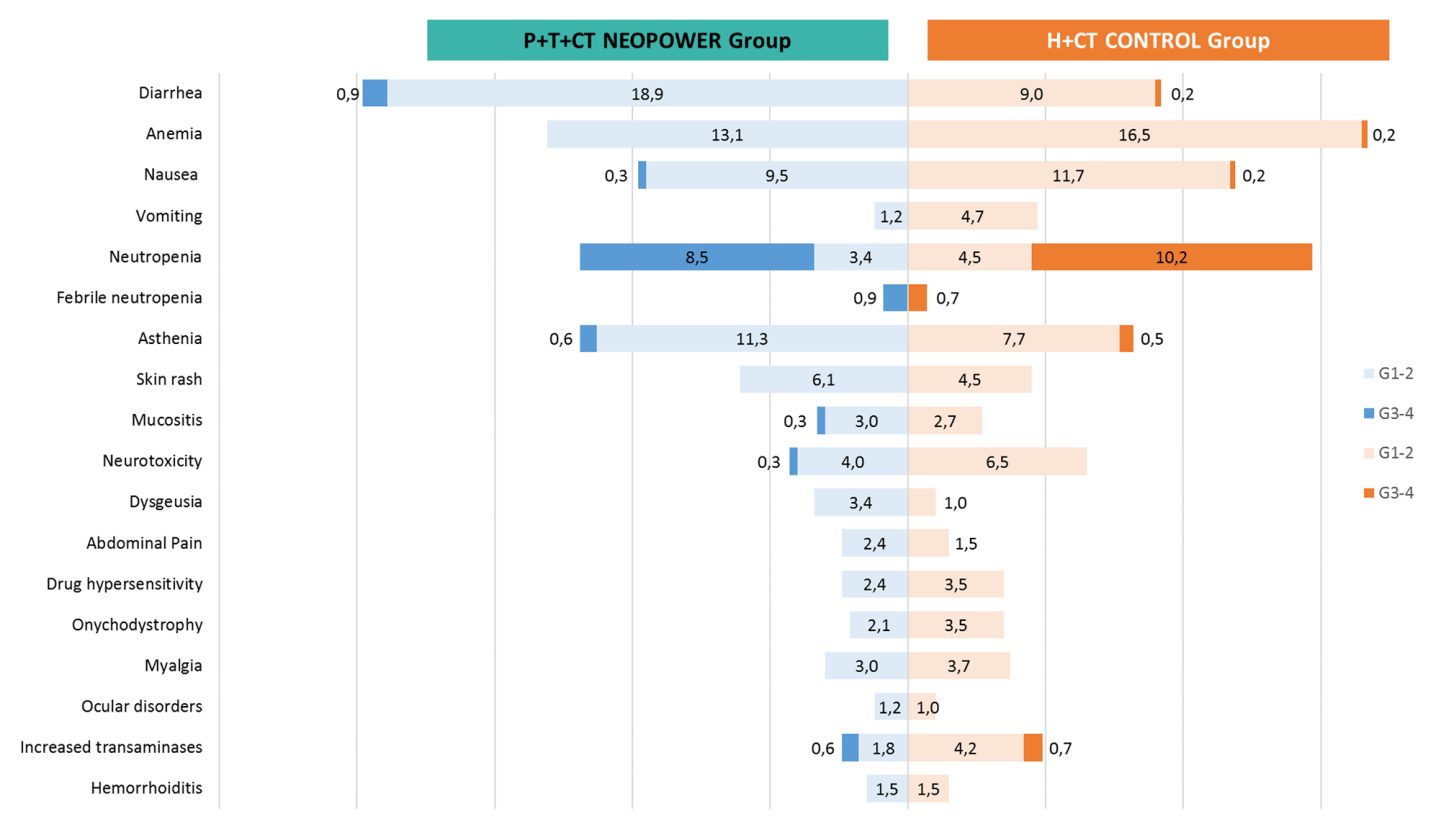
Overall AEs in *P* + H + CT and H + CT groups

In the *P* + H + CT cohort the most common AEs of any G were diarrhea 20%, anemia 13% and neutropenia 12%; conversely, in the control were anemia 17%, neutropenia 15% and nausea 12%. Figure 1 resume in detail incidence of AEs, by comparing the two treatment group.

The most frequent AE of G3-4 was neutropenia, 8.5% and 10% in the neopower and control cohort respectively. Of these, 6 events were febrile neutropenias, 3 for each group, 4 related to D. Twenty-three patients who were receiving D in the *P* + H + CT cohort, received granulocyte colony-stimulating factor (G-CSF) to prevent febrile neutropenia. None of the patients who had febrile neutropenia received prophylaxis with G-CSF.

Forty neurotoxicity (G1 = 77.5%, G2 = 20% and G3 = 2.5%) and 22 drug hypersensitivity events (G1 = 32% and G2 = 68%) were observed, 90% and 73% respectively associated with wPtx.

We recorded 3 serious adverse events (SAEs): 1 urinary tract infection (*P* + H + CT), 1 typhlitis and 1 sepsis (H + CT).

Drug related AEs led to a similar rate of dose reductions and drug-switch (D→wPtx) in both groups: 25% and 7% vs. 22% and 6% in the *P* + H + CT and control cohort respectively. In patients in the H + CT group more drug discontinuations were observed (9% vs. 2%).

Higher rates of diarrhea of anyG occurred in patients of *P* + H + CT group compared to control, 20% vs. 9%, less than 1% were diarrhea of G3-4.

Patients who received anthracyclines-containing regimens had higher rates of vomiting (4% vs. 1%) and nausea (13% vs. 7%).

### Cardiac safety analysis

**Fig. 2:**
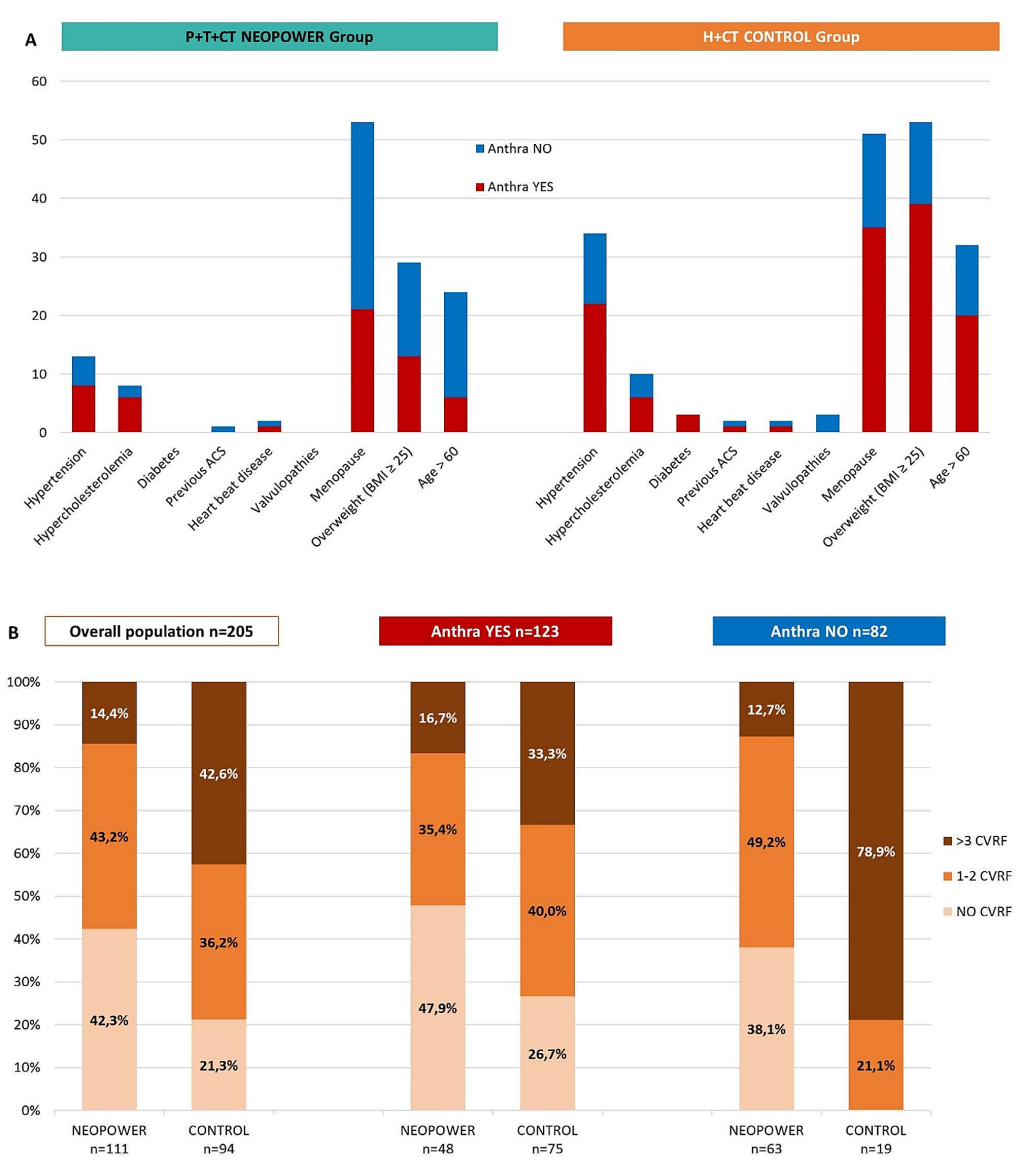
(**a**) Patients’ cardiovascular risk factors (CVRF) at diagnosis, distributed according to the treatment arm and the use of neoadjuvant anthracycline. (**b**) Number of CVRF per patient at diagnosis, distributed according to the treatment arm and the use of neoadjuvant anthracycline

**Fig. 3:**
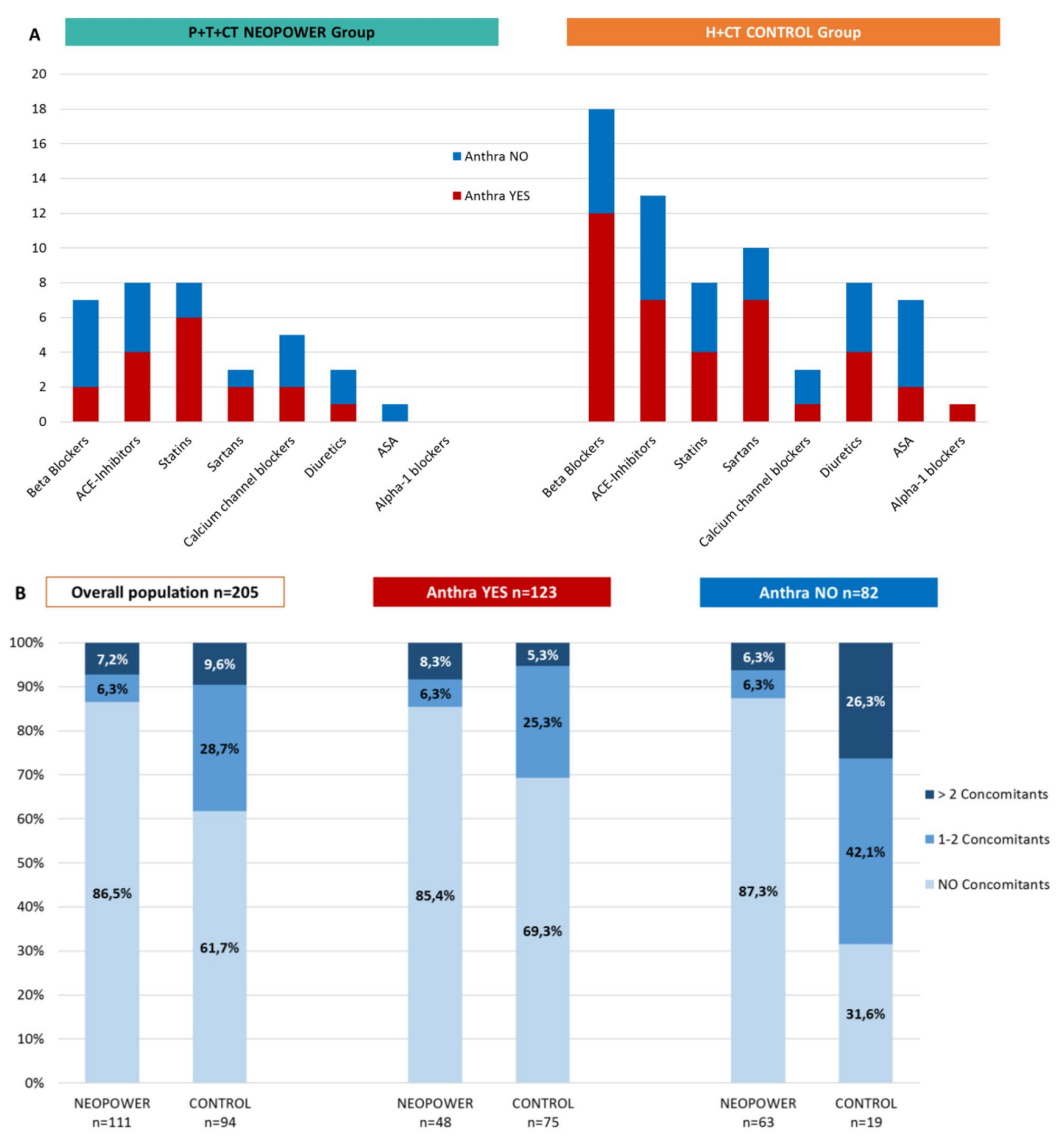
(**a**) Patients’ concomitant cardiovascular drugs at diagnosis, distributed according to the treatment arm and the use of neoadjuvant anthracycline. (**b**) Number of concomitant cardiovascular drugs per patient at diagnosis, distributed according to the treatment arm and the use of neoadjuvant anthracycline

At least two timepoints were needed to assess the cardiac safety and 205 patients were evaluable, 111 (54%) in the *P* + H + CT cohort. Data on any risk factors and concomitant drugs concerning the cardiovascular system were collected (Figs. 2 and 3).

The H + CT group had more patients with at least one CVRF (79%) and taking at least one concomitants at diagnosis (38%), compared to the neopower group, 58% at 13% respectively.

The median variation in LVEF pre- and after-neoadjuvant CT was − 5% in overall population and control group, -4% in neopower cohort.

**Fig. 4:**
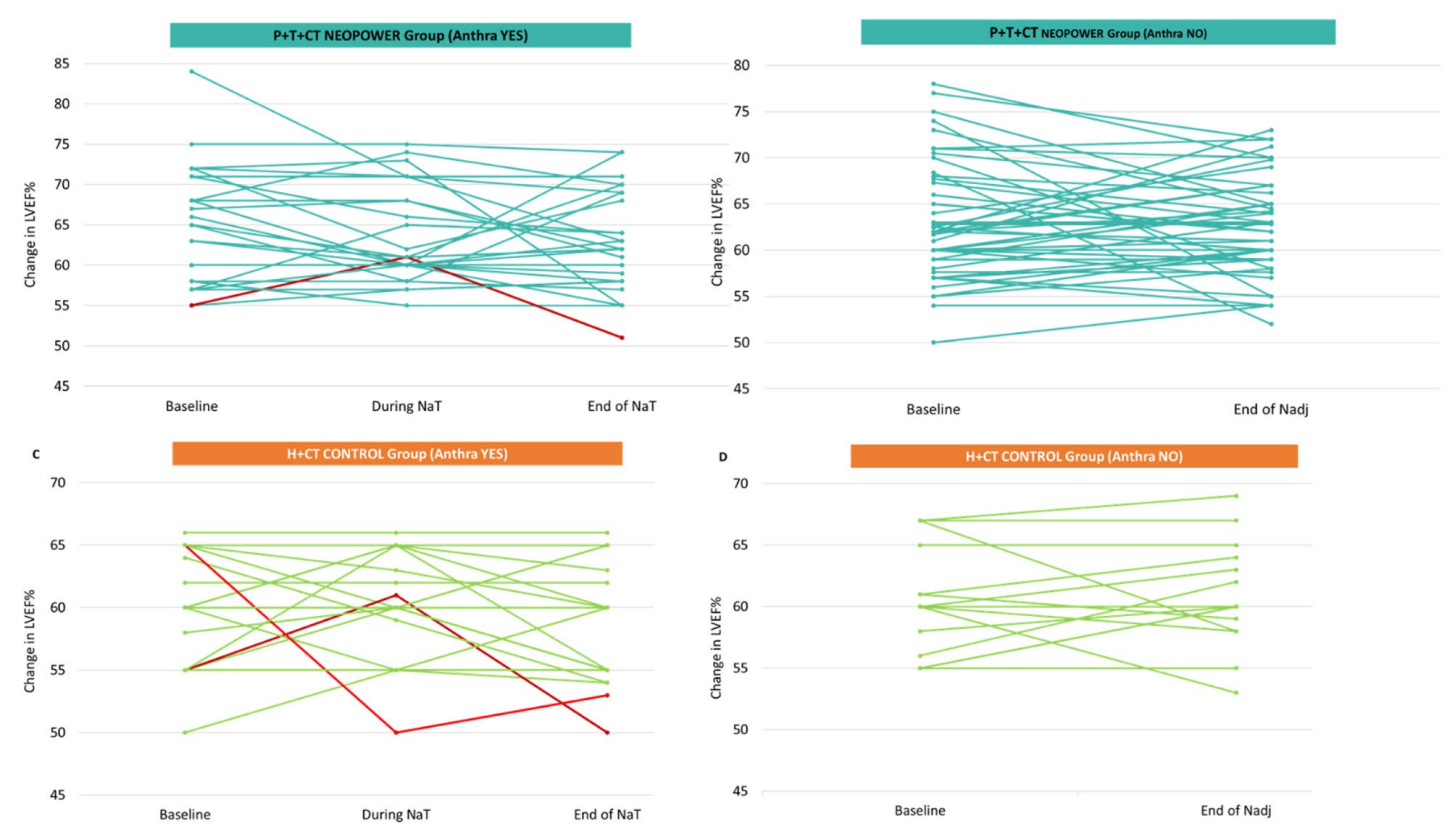
change in LVEF after neoadjuvant: (**a**) *P* + H + CT, Anthra YES; (**b**) *P* + H + CT, Anthra NO; (**c**) H + CT, Anthra YES; (**d**) H + CT, Anthra NO

After preoperative treatment, there were 3 (1.5%) significant LVEF reduction events, of which 2 (2%) occurred in the control group. All three patients were symptomatic and received anthracycline as part of neoadjuvant chemotherapy. Everyone had at least one CVRF, but only one of them already took concomitant medications. After temporary discontinuation of antineoplastics and cardioprotective therapy, we observed the recovery of LVEF in 2 patients. However, one of them required permanent treatments discontinuation. Figure 4 show LVEF trends during neoadjuvant treatment in each group, according to the use of neodiuvant anthracycline.

### pCR analysis

pCR analysis included 259 eligible patients. One patient in the neopower group prematurely stopped preoperative treatment because of adverse events and was not considered.

In overall population, pCR rates were 46% in *P* + H + CT cohort, slightly higher compared to 40% of the control group. The addition of P had no statistically significant correlation with pCR (OR = 1.24, 95%CI [0.76–2.03], *p* = 0.390), even after adjusting for imbalanced parameters between groups (OR = 1.63, 95%CI [0.92-3.00], *p* = 0.120).

**Fig. 5:**
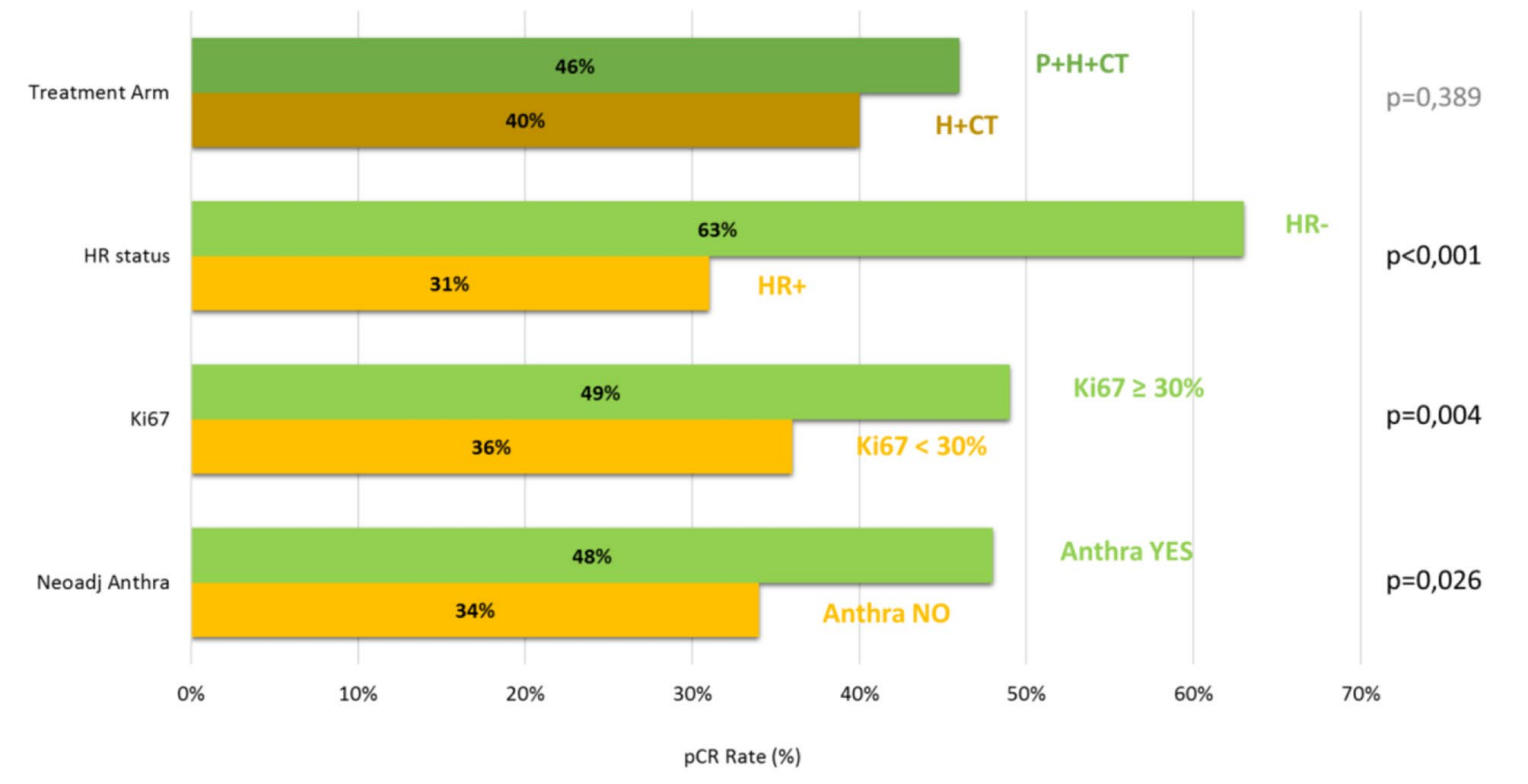
Differences in pCR rate in overall population according to statistically significant variables and treatment arm

At univariate analysis, HR negative (OR = 3.79, 95%CI [2.24–6.44], *p* < 0.001), estrogen – ER and progesterone receptors – PgR expressions (OR = 0.98, 95%CI [0.98–0.99], *p* < 0.001), Ki67 ≥ 30% (OR = 1.69, 95%CI [1.02–2.79], *p* = 0.040), the use of preoperative anthracyclines (OR = 1.81, 95%CI [1.07–3.07], *p* = 0.030) and neoadjuvant treatment duration (OR = 1.18, 95%CI [1.03–1.36], *p* = 0.020) resulted statistically related to pCR (Fig. 5).

By performing the same analysis in the subpopulation that received preoperative anthracyclines, results were similar: HR– disease (OR = 4.37, 95%CI [2.23–8.57], *p* < 0.001), ER (OR = 0.98, 95%CI [0.97–0.99], *p* < 0.001) and PgR (OR = 0.98, 95%CI [0.97–0.99], *p* = 0.003) expression, and Ki67 (OR = 1.02, 95%CI [1.00-1.05], *p* = 0.020) were statistically associated to pCR, P use was not (OR = 1.41, 95%CI [0.74–2.68], *p* = 0.300).

ER expression was found to be an independent factor associated with pCR in multivariate analysis, both in overall population (OR: 0.98, 95%CI [0.97–0.99], *p* = 0.005), and in the subpopulation receiving neoadjuvant anthracyclines (OR: 0.97, 95%CI [0.95–0.99], *p* = 0.001).

In the subgroup treated with anthracycline-free regimens, the addition of P was found to be statistically related to the pCR rate (OR = 3.05, 95%CI [0,94 − 9,95], *p* = 0.047), even in the analysis adjusted for unbalanced parameters between groups (OR = 5.65, 95%CI [1,04–30,65], *p* = 0.045). Once again HR– disease (OR = 3.31, 95%CI [1.34–8.14], *p* = 0.009), ER (OR = 0.99, 95%CI [0.98–0.99], *p* = 0.010) and PgR (OR = 0.98, 95%CI [0.97–0.99], *p* = 0.004) expression were associated with the pCR. None of them confirmed a statistically significant correlation in multivariate analysis. Table 4 shows the results of uni- and multi-variate analysis. Table 5 shows the analysis adjusted for imbalanced parameters between groups.

**Table 4:** Univariate and multivariate analysis of correlation between clinical-pathological features and pCR

VARIABLE	Overall population *n* = 259				Anthracycline YES *n* = 167				Anthracycline NO *n* = 92			
	Univariate analysis		Multivariate analysis		Univariate analysis		Multivariate analysis		Univariate analysis		Multivariate analysis	
	OR (95% CI)	*p*	OR (95% CI)	*p*	OR (95% CI)	*p*	OR (95% CI)	*p*	OR (95% CI)	*p*	OR (95% CI)	*p*
**Treatment Arm**												
- H + CT (Control)	Reference				Reference				Reference			
- *P* + H + CT (Neopower)	1,242 [0,759-2,033]	0,389			1,408 [0,739-2,682]	0,298			**3,054 [0,937-9,954]**	**0,047**	3,134 [0,916 − 10,722]	0,069
Age at diagnosis (years)*												
	0,996 [0,977-1,017]	0,730			0,999 [0,973-1,026]	0,955			1,001 [0,969-1,035]	0,934		
**Menopause**												
- No	Ref.				Ref.				Ref.			
- Yes	0,879 [0,527-1,464]	0,620			1,100 [0,586-2,065]	0,767			0,641 [0,261-1,576]	0,333		
**BMI**												
- < 25	Ref.				Ref.				Ref.			
- ≥ 25	0,619 [0,350-1,093]	0,097			0,586 [0,301-1,144]	0,116			0,837 [0,271-2,584]	0,757		
**PS ECOG**												
- 0	Ref.				Ref.				Ref.			
- 1	1,572 [0,678-3,643]	0,292			2,444 [0,851-7,023]	0,089			0,608 [0,110-3,348]	0,556		
**Clinical Lymphnode (cN)**												
- Negative	Ref.				Ref.				Ref.			
- Positive	0,908 [0,547-1,509]	0,711			0,835 [0,440-1,583]	0,580			0,902 [0,378-2,154]	0,817		
**Stage at diagnosis**												
- II	Ref.				Ref.				Ref.			
- III	0,598 [0,336-1,066]	0,077			0,540 [0,269-1,083]	0,079			0,675 [0,234-1,944]	0,459		
**Grading**												
- 2	Ref.				Ref.				Ref.			
- 3	1,725 [0,893-3,332]	0,098			1,369 [0,621-3,015]	0,434			2,992 [0,788 − 11,365]	0,082		
ER (%)*												
	**0,983 [0,977-0,989]**	**< 0,001**	**0,980 [0,967-0,994]**	**0,005**	**0,979 [0,971-0,987]**	**< 0,001**	**0,966 [0,946-0,986]**	**0,001**	**0,988 [0,978-0,998]**	**0,013**	1,004 [0,982-1,026]	0,746
PgR (%)*												
	**0,985 [0,978-0,993]**	**< 0,001**	0,999 [0,989-1,010]	0,915	**0,986 [0,977-0,996]**	**0,003**	1,006 [0,992-1,020]	0,430	**0,980 [0,965-0,995]**	**0,004**	0,982 [0,962-1,003]	0,091
**HR status**												
- Negative (HR-)	Ref.		Ref.		Ref.		Ref.		Ref.		Ref.	
- Positive (HR+)	**0,263 [0,155-0,447]**	**< 0,001**	1,245 [0,399-3,885]	0,705	**0,229 [0,117-0,448]**	**< 0,001**	2,819 [0,526 − 15,099]	0,226	**0,302 [0,123-0,744]**	**0,008**	0,502 [0,077 − 3,267]	0,471
**Ki67**												
- < 30%	Ref.		Ref.		Ref.				Ref.			
- ≥ 30%	**1,688 [1,022 − 2,790]**	**0,040**	1,416 [0,814-2,464]	0,219	1,806 [0,972-3,356]	0,060			1,782 [0,720-4,409]	0,205		
NaT duration (Cycles)*												
	**1,181 [1,026 − 1,360]**	**0,018**	1,320 [0,891-1,956]	0,166	1,149 [0,632-2,088]	0,645			1,146 [0,764-1,719]	0,509		
**NaT duration cutoff**												
- ≤ 4 cycles	Ref.				NA				Ref.			
- 5–8 cycles	1,584 [0,894-2,805]	0,111			NA				0,820 [0,297-2,268]	0,701		
**Neoadjuvant Anthra**												
- No	Ref.		Ref.		NA				NA			
- Yes	**1,809 [1,067 − 3,069]**	**0,026**	0,800 [0,187-3,425]	0,763	NA				NA			
**Time to surgery**												
- ≤ 28 days	Ref.				Ref.				Ref.			
- > 28 days	1,015 [0,617-1,670]	0,954			0,823 [0,445-1,523]	0,534			1,528 [0,638-3,656]	0,339		

*for continuous variables the OR refers to increases of 1 unit of measurement (indicated in brackets)

**Table 5:** Correlation analysis to pCR adjusted for features imbalanced between arms

VARIABLE	Overall population *n* = 259		Anthra YES *n* = 167		Anthra NO *n* = 92	
	OR (95% CI)	*p*	OR (95% CI)	*p*	OR (95% CI)	*p*
**Treatment Arm**						
- H + CT (Control)	Reference		Reference		Reference	
- *P* + H + CT (Neopower)	1,66 [0,92 − 3,0]	0,093	1,39 [0,57 − 3,43]	0,468	**5,65 [1,04–30,65]**	**0,045**
**Stage at diagnosis**						
- II	Ref.		Ref.		NA	
- III	0,60 [0,33 − 1,11]	0,103	0,49 [0,20 − 1,19]	0,114	NA	
**Ki67**						
- < 30%	Ref.		NA		Ref.	
- ≥ 30%	**1,71 [1,01–2,91]**	**0,047**	NA		1,39 [0,50 − 3,90]	0,528
NaT duration (Cycles)*						
	1,20 [0,82 − 1,74]	0,342	NA		0,96 [0,58 − 1,05]	0,860
**Neoadjuvant Anthra**						
- No	Ref.		NA		NA	
- Yes	1,25 [0,30 − 5,27]	0,759	NA		NA	
**Time to surgery**						
- ≤ 28 days	Ref.		NA		Ref.	
- > 28 days	0,89 [0,53 − 1,52]	0,103	NA		1,36 [0,52 − 3,57]	0,535

### Survival analysis

Median follow up duration was 36,5 [range 5–77] and 71 [10–176] months in neopower and control group respectively.

DRFS analysis included 257 of the 260 eligible patients. Three patients were excluded due to incomplete data. Twentyone distant relapse events occurred: 9 in the *P* + H + CT cohort and 12 in the control; 3-years DRFS rate was 89.7% (95%CI 82.6–96.8%) and 93.8 (95%CI 89.7–97.9%) respectively.

OS analysis included 258 of the 260 eligible patients. A total of 17 deaths occurred: 3 in neopower and 14 in the control group; 3-years OS rate was 100% and 96.1 (95%CI 92.7–99.5%) respectively.

**Fig. 6:**
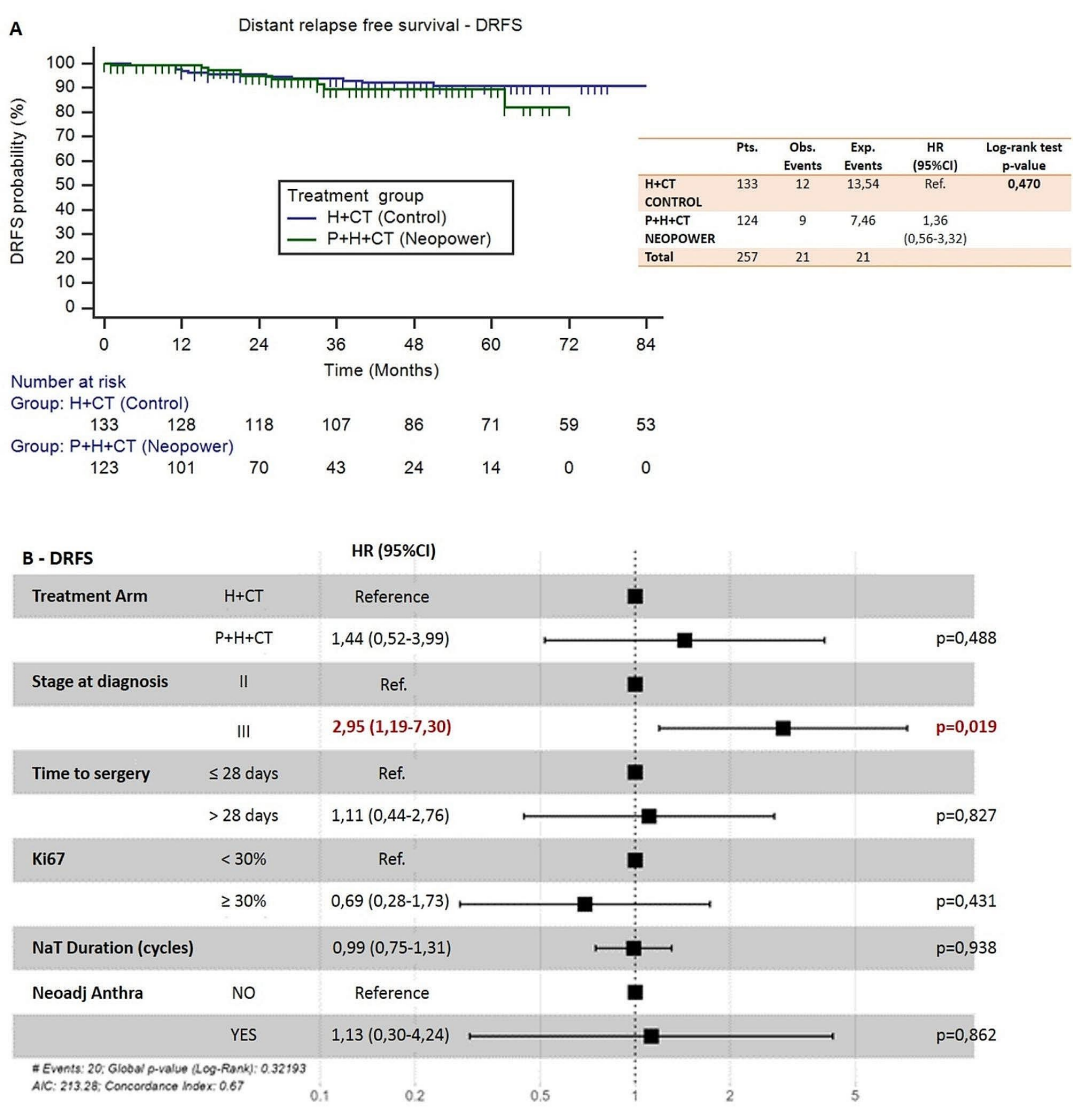
(**a**) Kaplan Meier curves for distant relapse free survival – DRFS; (**b**) Forest plot representing Cox proportional hazard model adjusted for unbalanced parameters related to DRFS (Neopower vs. Control)

**Fig. 7:**
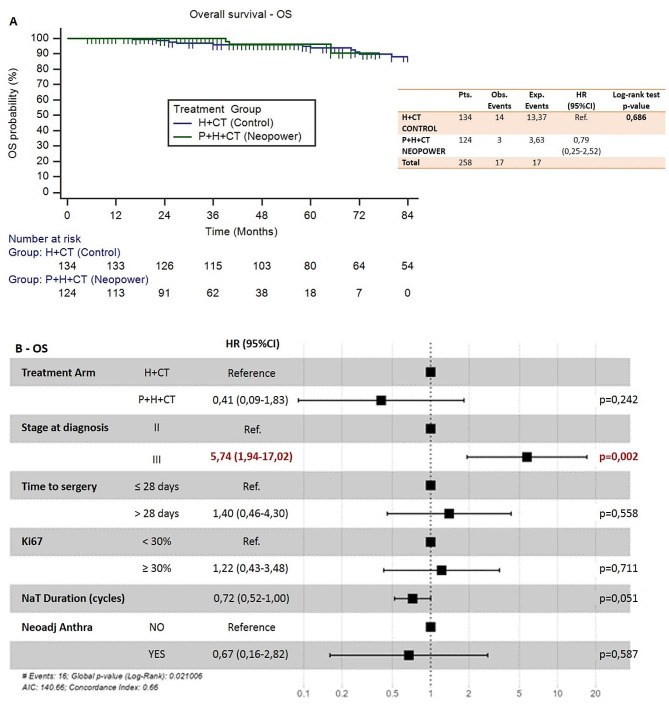
(**a**) Kaplan Meier curves for overall survival - OS; (**b**) Forest plot representing Cox proportional hazard model adjusted for unbalanced parameters related to OS (Neopower vs. Control)

The Cox proportional hazard model, adjusted for unbalanced parameters between groups, showed that the addition of P was not statistically related to an improvement in DRFS (HR = 1.44, 95%CI 0.52–3.99, *p* = 0.490) and OS (HR = 0.41, 95%CI 0.09–1.83, *p* = 0.240). In this model, stage III at diagnosis was the only prognostic variable correlated with statistical significance to survival (HR = 2.95, 95%CI 1.19–7.30, *p* = 0.019 for DRFS; HR 5.74, 95%CI 1.94–17.02, *p* = 0.002 for OS). Figures 6 and 7 shows the Kaplan Meier curves and the forest plots that represent these relationships for DRFS and OS respectively.

The same analysis was performed by comparing patients who achieved the pCR and those who had residual invasive disease after preoperative treatment (no-pCR).

**Fig. 8:**
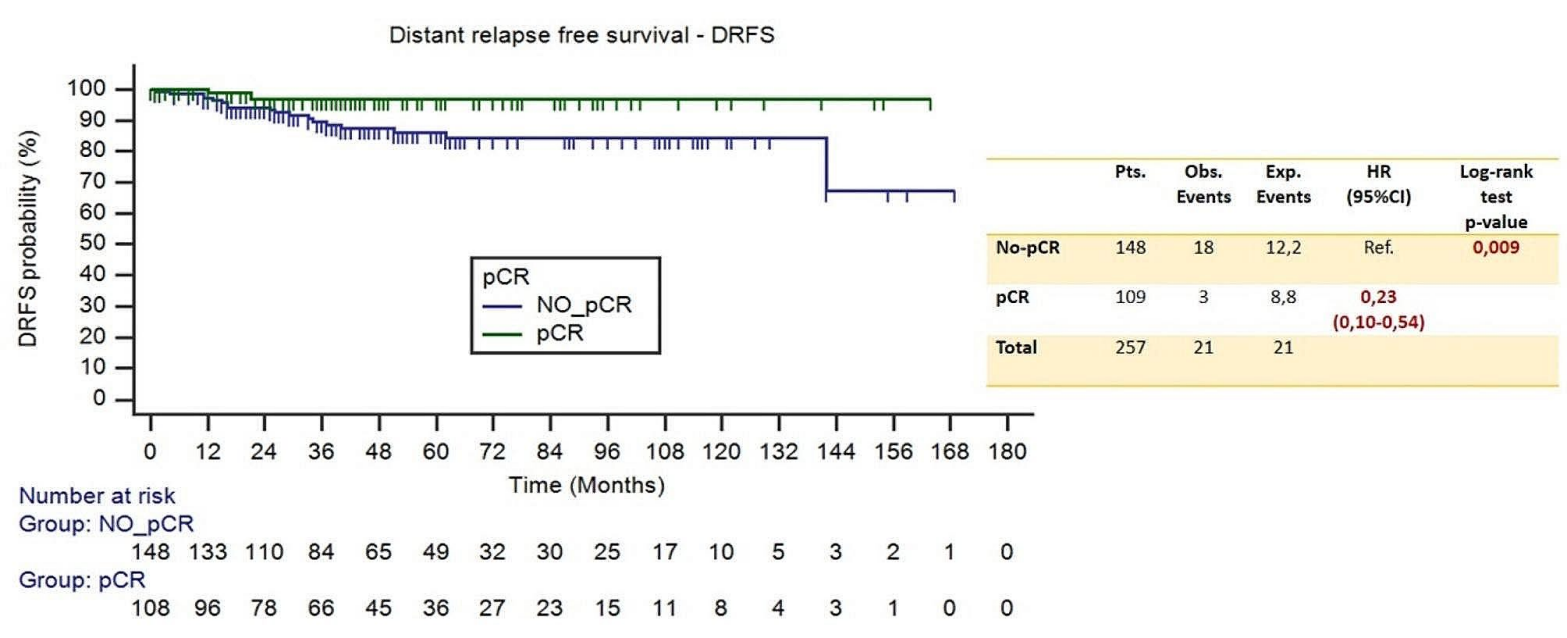
Kaplan Meier curves for distant relapse free survival – DRFS (pCR vs. no-pCR)

**Fig. 9:**
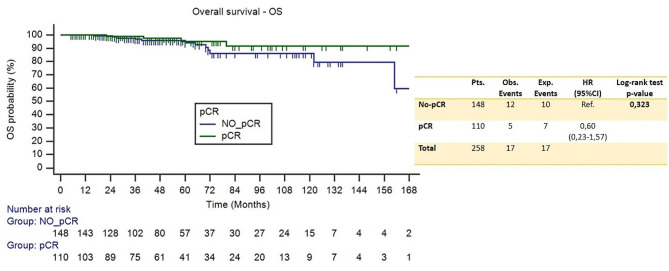
Kaplan Meier curves for overall survival – OS (pCR vs. no-pCR)

Of the 21 distant relapse events and 17 deaths, 18 and 12 respectively occurred in the no-pCR group. Compared to those with residual invasive disease, patients who achieve pCR had a significant increase in DRFS (HR 0.23, 95%CI [0.10–0.54], *p* = 0.009), but not in OS (HR 0.60, 95%CI [0.23–1.57], *p* = 0.323). Figures 8 and 9 shows the Kaplan Meier curves that represent these relationships for DRFS and OS respectively.

## Discussion

Based on the results shown, the NeoPowER study reaches its primary endpoint, safety: the addition of P to H and CT, as neoadjuvant treatment in stage II-III HER2 + BC patients, is confirmed to be safe. The AEs rate of G3-4 (about 12%) and toxicity profile, including cardiac events that occurred in about 2% of patients, were overlapping in both groups. Diarrhea was the only AE significantly more frequent in the *P* + H + CT group than control (20% vs. 9%), although of G ≤ 2 in almost all cases (only 3 out of 65 events were of G3).

It should be noted that even the rate of severe neutropenias and febrile neutropenias were similar between groups, despite the wider use of D in the neopower cohort compared to control (63% vs. 6%). This data is probably related to the use of G-CSF as primary prevention for febrile neutropenia. All patients who received this prophylaxis belonged to the *P* + H + CT cohort, none developed febrile neutropenia, 3 of them developed neutropenia of G3 between the sixth and eighth course of preoperative treatment, in the anthracycline phase.

Interpretation of results is more complex for secondary endpoints. In patients who received anthracycline-free regimens, the addition of P correlated significantly with pCR. Those receiving H and taxane alone achieved the lowest rates of pCR (17% vs. ≥ 40% adding P and/or anthracyclines), despite more favorable clinical-pathological factors than the overall population (G3 68% vs. 78%, median Ki67 25% vs. 30%). To date, neoadjuvant treatment with single cytotoxic agent (taxane) and H is to be considered sub-optimal in patients with stage II-III HER2-positive breast cancer.

However in our study, the addition of P was not statistically related to pCR in overall population. Conversely, in univariate analysis, use of neadjuvant anthracyclines and duration of preoperative treatment were related to pCR with statistical significance, as well as HR negative and high proliferative index (Ki67 ≥ 30%) disease, these findings wer not confirmed at multivariate analysis.

While not achieving statistical significance on this secondary endpoint, some considerations need to be made. Although we observed a limited use of anthracycline (44% vs. 83%) and a consequent shorter median duration of neoadjuvant treatment (119 vs. 153 days) in *P* + H + CT cohort compared to the control, the first saw a numerically higher pCR rate (46% vs. 40%). In our population all the three cardiac events occurred in patients who received both anthracyclines and HER2-inhibitors, even if in a sequential strategy. Moreover, the use of anthracyclines were related to a higher rate of nausea and vomiting, well manageable AEs, but which could significantly affect the patient’s quality of life. These data should be taken into account, especially considering evidence from randomized controlled trials, such as TRYPHAENA [8, 9] and TRAIN-2 [11, 12], which showed that HER2 dual blockade associated with anthra-free chemotherapy (carboplatin-taxane) compared to anthracycline-contaning regimens, allows to achieve similar pCR rates and long-term outcomes with a more favourable toxicity profile.

The overall pCR rate observed in the neopower group (46%) was lower than those obtained in other real world experiences (range 51–68%) carried out by several authors in the same setting [13–19]. The heterogeneity of cytotoxic treatments associated to HER2 dual blockade in our study may have contributed to the difference observed. In the *P* + H + CT group, patients treated with neoadjuvant anthracycline obtained a pCR rate of 54%, similar to other real-world studies. The pCR rate dropped to 39% in patients who received an anthra-free regimen. Of these, 70% received 4 courses of *P* + H + taxane and 48% adjuvant anthracyclines. This is the same treatment scheme used in the NeoSphere trial, in which patients in the *P* + H + Docetaxel arm achieved a comparable “total pCR” rate (complete pathological response on breast and axillary lymph-nodes) of 39% [6]. Thus, over two-third of this subpopulation received shorter neoadjuvant CT (4 cycles) than used in both current clinical practice and most of the previously mentioned real world and clinical trials (range 6–9 cycles). Note that the treatment scheme of the NeoSphere trial has been the reference in our centers for some time. It should also be noted that, in our study, 85% of patients received this treatment scheme between 2016 and 2019, prior to the results of the KATHERINE trial [20] and the availability of adjuvant trastuzumab-emtansine (TDM1) for patients with residual invasive disease after NaT. This change in clinical practice, together with the increasing amount of data showing better results in pcr rate using longer preoperative treatments, probably led clinicians to anticipate anthracycline more frequently in the neoadjuvant phase, diverging from the scheme used in the NeoSphere trial. Finally, only 1 out of 69 patients received carboplatin associated to *P* + H and taxanes. In the period analyzed, it was not our daily clinical practice to add carboplatin instead of anthracyclines for the pre-operative treatment of these patients. The addition of carboplatin to anthra-free regimens and double HER2 blockade has been correlated with a numerical increase in pCR rates [21–23] and event-free survival (EFS), at the expense of an increased incidence of thrombocytopenia of G > 2 (13%) [24]. It could be therefore assumed that both the shorter duration of the NaT and the lack of carboplatin in the anthra-free regimens contributed to different results compared to similar real world experiences.

Although pCR retains a leading role as a surrogate for long-term efficacy results in neoadjuvant studies, the “invasive residual disease” may be a limited concept today. In other settings, many experiences showed that preoperative treatments can have very different long-term outcomes on the individual patient, depending on the burden of invasive residual disease [25]. For this reason, the use of residual cancer burden (RCB) to stratify individual risk is becoming increasingly widespread [26]. The efforts of future research should not be limited to modulating the pre-operative treatment, which represents a crucial phase for the treatment of these patients, but also the post-operative according to this risk, in order to get closer and closer to the concept of personalized medicine. Some ongoing trials are heading in this direction (CompassHER2 pCR - NCT04266249–2020/02/12, CompassHER2 RD - NCT04457596–2020/07/07, Decrescendo - NCT04675827–2020/12/19).

The strengths of NeoPowER study were the multicentric design, which allowed to reach an adequate sample size despite the objective difficulties to use neoadjuvant P in Italy before november 2023; and the comparison with the control arm, although indirect, performed only in a limited number of other real-world experiences. A limitation was the retrospective design, involving a time frame during which clinical practice changed as previously argued; this period was particularly long for the control group (2007–2021), while it was shorter for the NEOPOWER group (2016–2022). This was related to the decision to include in the historical control group only patients treated at the Modena centre: a longer time frame was necessary to obtain an adequate sample size, while being aware of the potential bias this could have introduced. For an in-depth discussion of this topic, see the supplementary Table S1. The heterogeneity of chemotherapy schemes associated to HER2 dual blockade were also a limitation of the study. These factors may have affected efficacy outcomes.

## Conclusions

NeoPowER real world trial confirm that adding neoadjuvant P to H and chemotherapy is safe, even when compared to H + CT alone. With the exception of diarrhea, toxicity profile does not differ between the two groups.

Moreover P doesn’t increase the cardiotoxicity when added to H + CT, nevertheless in our population all cardiac events occurred in patients who received anthracycline-containing regimens.

The study did not show a statistically significant difference in pCR rates in patients receiving neoadjuvant *P* + H + CT, when compared to H + CT. HR negative disease, Ki67 ≥ 30%, the use of preoperative anthracyclines and neoadjuvant treatment duration resulted statistically related to pCR rate.

The study did not show a statistically significant correlation between the addition of P and long-term outcomes (DRFS and OS). Residual invasive disease remains a negative prognostic factor.

It could be assumed that both the shorter duration of NaT and the lack of carboplatin in the anthra-free regimens received by most patients in the NEOPOWER group, contributed to the failure to reach the secondary endpoints of efficacy of our study.

### Electronic supplementary material

Below is the link to the electronic supplementary material.

Supplementary Material 1

Supplementary Material 2

## Data Availability

The data that support the findings of this study are not openly available due to reasons of sensitivity and are available from the corresponding author upon reasonable request. Data are located in controlled access data storage at University Hospital of Modena.

## Abbreviations

P: Pertuzumab
H: Trastuzumab
CT: Chemotherapy
NaT: Neoadjuvant treatment
pCR: Pathological complete response
HER2: Human epidermal growth factor 2
HER2+: HER2 positive
HER2−: HER2 negative
EFS: Event-free survival
OS: Overall survival
PFS: Progression free survival
DFS: Disease free survival
eBC: Early breast cancer
ECOG: Eastern Cooperative Oncology Group
IHC: Immunohistochemistry
ISH: In situ hybridization
CTCAE: Common Terminology Criteria for Adverse Events
ITC: Isolated tumor cell
DRFS: Distant relapse free survival
ER: Estrogen receptors
PgR: Progesterone receptors
LVEF: Left ventricular ejection fraction
IQR: Interquartile range
SD: Standard deviation
HR: Hazard ratios
D: Docetaxel
wptx: Weekly paclitaxel
CBDCA: Carboplatinum
Anthra: Anthracycline
EC: Epirubicin-Cyclophosphamide
TDM1: Trastuzumab-emtansine
SLNB: Sentinel Node Biopsy
ALND: Axillary lymph node dissections
AE(s): Adverse events
G: Grade
SAEs: Serious adverse events
CVRF: Cardiovascular risk factors
OR: Odds ratio
HR*: Hormone receptors
