# Supplementary material for: Safety and efficacy analysis of neoadjuvant pertuzumab, trastuzumab and standard chemotherapy for HER2–positive early breast cancer: real–world data from NeoPowER study
Source: BMC Cancer. 2024 Jun 15;24:735. doi: 10.1186/s12885-024-12506-0 (PMC11179289; doi:10.1186/s12885-024-12506-0)
Supplement: Supplementary file 2 — Supplementary Material 2 [file 12885_2024_12506_MOESM2_ESM.docx]

**Supplemetary**

A section has been added in which we analyse the characteristics of patients in the H+CT group treated at the Modena centre between 2016 and 2021, when pertuzumab also became available.

| **Patients treated with H+CT from 2016 and 2021** | | | | |
| --- | --- | --- | --- | --- |
| **Year** | **N° of pts** | **Stage II vs III** | **Ki67 <30 vs ≥ 30** | **CVRF 0 vs ≥ 1** |
| 2016 | 12 | 9 vs 3 | 10 vs 2 | 7 vs 3 |
| 2017 | 13 | 8 vs 5 | 9 vs 4 | 5 vs 8 |
| 2018 | 20 | 17 vs 3 | 13 vs 7 | 8 vs 12 |
| 2019 | 8 | 6 vs 2 | 6 vs 2 | 7 vs 1 |
| 2020 | 9 | 9 vs 0 | 6 vs 3 | 8 vs 1 |
| 2021 | 3 | 2 vs 1 | 2 vs 1 | 2 vs 1 |

The table presents a stratification of patients according to the year of treatment. Disease characteristics representative of burden and aggressiveness (stage and Ki67) and cardiovascular risk factors present at diagnosis are shown. Despite the availability of pertuzumab, these patients did not receive anti-HER2 double blockade. However, the table indicates that this treatment was also prescribed to patients with a higher disease burden or who did not have cardiovascular risk factors at diagnosis to contraindicate the use of anti-HER2 double blockade. It can be assumed that the characteristics of the population under consideration are similar to those of the control group as a whole, and therefore, there is no potential for bias.

The reasons why patients were treated without pertuzumab between 2016 and 2021 may be different. The introduction of the drug in the neoadjuvant setting was not immediate in Italy; rather, it took years. As discussed in the introduction, the Italian healthcare system only made the antibody reimbursable with this indication from November 2023. Prior to this date, in order to utilise the drug, it was necessary to submit a request for nominal use for each individual patient to the ethics committee, awaiting a response. In the majority of cases, requests were accepted, although in some instances, patients were unable to wait for a response. It is likely that this procedure became more 'automatic' over time, in part thanks to the growing body of literature supporting the use of double blockade as neoadjuvant treatment. The number of patients treated with H+CT exhibited a gradual decline from 2019 onwards, as illustrated in the table.
